# Supplementary material for: Strain control of hybridization between dark and localized excitons in a 2D semiconductor
Source: Nat Commun. 2022 Dec 12;13:7691. doi: 10.1038/s41467-022-35352-9 (PMC9744834; doi:10.1038/s41467-022-35352-9)
Supplement: Supplementary file 1 — Supplementary Information [file 41467_2022_35352_MOESM1_ESM.pdf]

Strain control of hybridization between dark and localized excitons in a 2D semiconductor
Supplementary Information

Pablo Hernández López*[1†], Sebastian Heeg*[1†], Christoph Schattauer[3], Sviatoslav Kovalchuk[2], Abhijeet Kumar[2], Douglas J. Bock[2], Jan N. Kirchhof[2], Bianca Höfer[2], Kyrylo Greben[2], Denis Yagodkin[2], Lukas Linhart[3], Florian Libisch[3], Kirill I. Bolotin[2†]

* These authors contributed to the work equally

[1] Institut für Physik and IRIS Adlershof, Humboldt Universität zu Berlin, 12489 Berlin, Germany

[2] Physics Department, Freie Universität Berlin, 14195 Berlin, Germany

[3] Vienna University of Technology, 1040 Vienna, Austria

[†] Corresponding authors

SUPPLEMENTARY NOTE 1: POSSIBLE ARTEFACTS

Several phenomena can contribute to gate-dependent changes in PL intensity and potentially produce signatures similar to the ones ascribed in the main text to hybridization between bright and localized excitons. Here, we discuss such artefacts and show that none of them can explain our observations (Figs. 3-5 of the main text).

- **Fermi level changes.** Changes of the gate voltage V_G produce changes in the Fermi level of our device in addition to the changes in strain (see Eq. 1 and 2 of the main text). In principle, changes in the Fermi level affect photoluminescence spectra. However, the gradual increase in E_F only produces a Stokes shift in the energetic positions of trion vs. neutral exciton and a gradual decrease in the intensity of all excitonic peaks due to phase-space filling effects and screening [1]. In contrast, our experimental data exhibit sharp peaks and avoided crossing signatures at specific strain values.
- **Interference effects.** The space between the WSe_2 membrane and a reflective Si backgate forms an optical cavity where the excitation laser beam interferes with itself. This interference results in a spatial modulation in the laser intensity inside the cavity. When the membrane actuated by V_G deflects, its center moves with respect to the interference pattern leading to changes in absorption. Changes in absorption, in turn, affect PL emission, so the overall PL intensity ultimately reflects the spatial modulation of the laser intensity. To carefully analyze the contribution of this effect we carried out optical interferometry and photoluminescence excitation measurements described in Supplementary Fig. 9. These measurements indicate that the contribution of the interference to V_G-dependent changes in PL intensity is small ($<$ 30 % of the overall PL signal seen in Figs. 3-5 at zero strain) and does not significantly affect our observations (the change of the overall PL signal intensity we attribute to hybridization is about a factor of six compared to PL at zero strain).
- **Changes in absorption.** As strain is applied to the WSe_2 monolayer, its optical absorption spectrum gradually shifts with respect to the excitation laser kept at $\lambda = 532\,\text{nm}$. Changes in absorption, in turn, affect the overall brightness of photoluminescence. For the absorption spectrum of monolayer WSe_2, however, our excitation energy of 2.33 eV lies well above the two main excitonic resonances A and B [2]. Using available data, we estimate that at 100 K, the temperature of measurements in Figs. 3 and 4, absorption should increase 30% from its unstrained value between 0 and 1.5% strain. It then decreases slowly as the strain is increased above $> 1.5\,\%$. In contrast, our experimentally observed PL intensities do not reflect these changes in absorption. The PL intensity increases 6 times at 0.75% compared to the unstrained value, decreases and then increases again 2 times at 2.4%, see Figure 3 of the main text. The difference between the PL emission behaviour with strain and the expected changes in absorption for the same strain values allow us to dismiss absorption as the main cause of the experimental features observed in Figs. 3,4 and 5 of the main text.
- **Strain-dependent scattering between K- and Q-valleys.** Strain-dependent scattering between K- and Q-valleys of WSe_2 has been previously invoked to explain strain-dependent changes of the PL intensity [3, 4]. It was argued that the strain increases the energy separation between the K- and Q-valleys thereby suppressing K-Q intervalley scattering. This, in turn, reduces the linewidth of the bright neutral exciton [3]. We do observe a corresponding reduction of the bright neutral exciton linewidth at room temperature with increasing strain up until 0.75 %. However, for strain $> 0.75\,\%$ the observed linewidth of the emission increases as PL splits in two bands (see Figure 5c in the main text). Continuous distancing of the K- and Q- valleys can explain neither the two PL resonances arising at different strain values (Figs. 3-5 of the main text) nor the splitting of the PL emission in two bands (Fig. 3). We therefore conclude that strain-induced changes in the relative energies of the K- and Q-valleys cannot account for the observation of hybridized peaks and other features seen in our experiments.

To summarize, none of the phenomena discussed above can contribute to the non-monotonic changes in PL spectra seen in Figs. 3-5 of the main text.

It is also worth discussing several differences between experiments and the theory (Fig. 4 of the main text). First, in experiments, we observe splitting of the peak X_d^0 into two peaks after the hybridization with $D1$ is reached at about 1% strain, with one peak continuing to shift linearly and another exhibiting weaker strain dependence (see also Fig. 3d in the main text). This behaviour signals avoided crossing between the states X_d^0 and $D1$, and was also predicted by the tight-binding model, but with a smaller amplitude. The absence of the defect state D_1 at strain values above the hybridization regime in our calculations is an artefact of the finite number of single-particle states

we calculate: as many conduction band states move down with strain, the defect state is effectively pushed out of the subspace we evaluate. We have checked numerically the effect of increasing the subspace size and find no change in the states already previously converged in the smaller subspace, as well as only minimal changes in the outgoing PL tail for Supplementary Fig. 14(a). Due to the associated numerical cost we thus refrain from increasing the subspace size. The more localized nature of the region of increased PL in strain in theory compared to experiments may indicate that excitonic states other than X_d^0 hybridize with $D1, D2$. Finally, the relatively high brightness of $D1, D2$ before reaching the hybridization in simulations compared to theory is likely related to the size of our defect supercell, that overestimates the defect concentration, as well as the absence of funneling effects which would further increase the maximum at the crossing point.

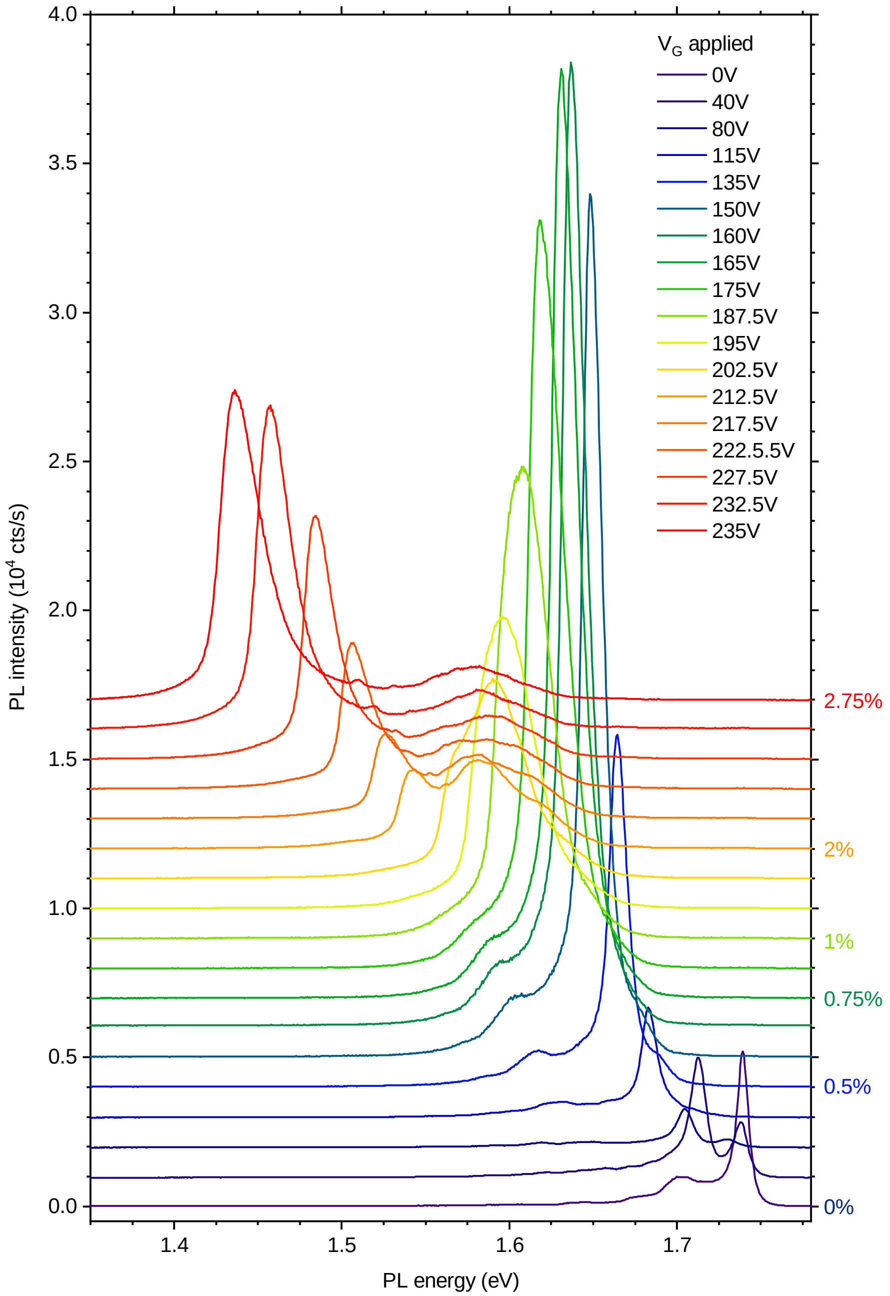

SUPPLEMENTARY FIG. 1. **Strain response of excitonic states in WSe_2 at 100 K.** PL spectra of the suspended WSe_2 monolayer (device 1) at $T = 100\,\mathrm{K}$ and positive V_G reproduced from Fig. 3b of the main text. Here the spectra are offset for clarity to allow for tracking individual peaks as a function of gate voltage / strain. The strain values assigned to selected spectra are shown next to the corresponding spectra.

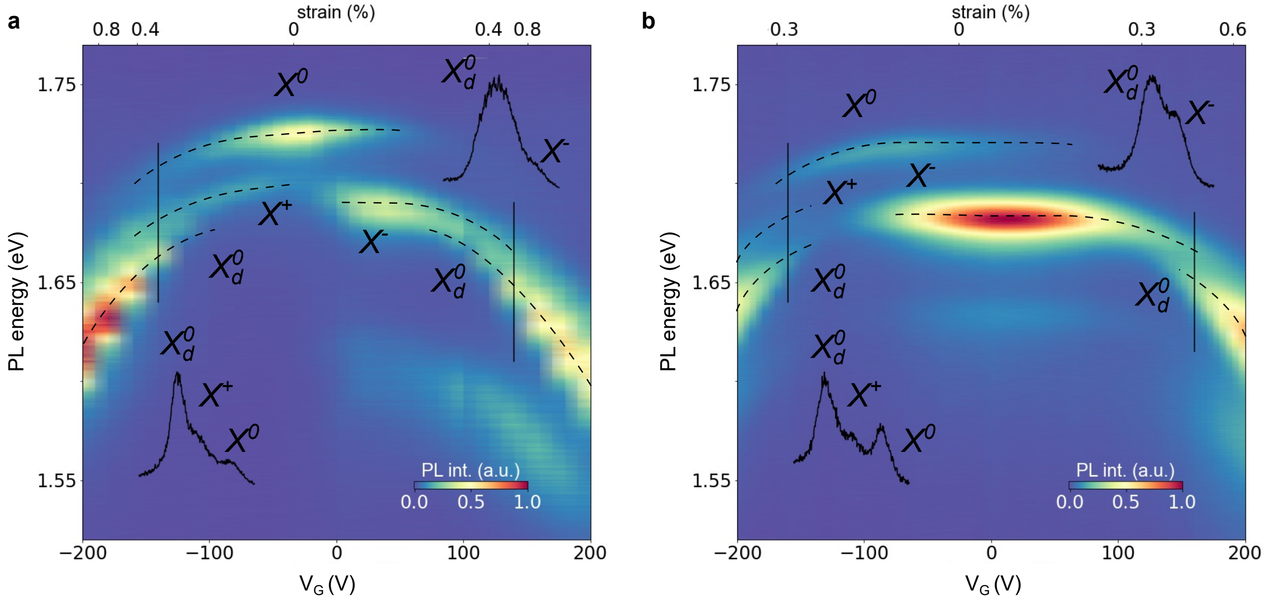

SUPPLEMENTARY FIG. 2. **Decoupling doping- and strain-induced effects in gating experiments.** As we note in the main text, our electrostatic straining technique implies that the carrier density in our devices changes together with strain upon gating. Here we experimentally disentangle the effects of doping and strain in our gated devices by comparing two PL intensity maps vs. V_G measured on the same device with different intrinsic doping levels. (a) and (b) show PL vs. V_G on device 2 at 77 K from two measurements performed three months apart. Excitonic species are marked with dashed lines and labeled. Individual spectra extracted from the black solid lines are shown in insets nearby. Degradation of monolayer WSe_2 over time due to chemical instability varies the defect and initial charge carrier concentrations of the samples. The PL spectrum at V_G=0 is dominated in (a) by neutral excitons and in (b) by negative trions. We therefore conclude that the sample is close to charge neutrality in (a) and initially electron doped in (b). The main doping-related effect, the redistribution of oscillator strength between neutral exciton and trions, happens symmetrically for negative and positive voltages around the charge neutrality point, V_G=0 in (a) and $V_G \approx$ -100V in (b). The brightening of the dark neutral exciton (X_d^0), however, happens at roughly the same energy for the two V_G polarities in both (a) and (b) because it is caused by the strain-driven hybridization of X_d^0 with a defect state when their energetically approach. We thus decouple strain and doping effects based on the fact that doping-induced changes in the PL depend on the polarity of V_G whereas strain-induced changes are symmetrical with respect to the sign of V_G.

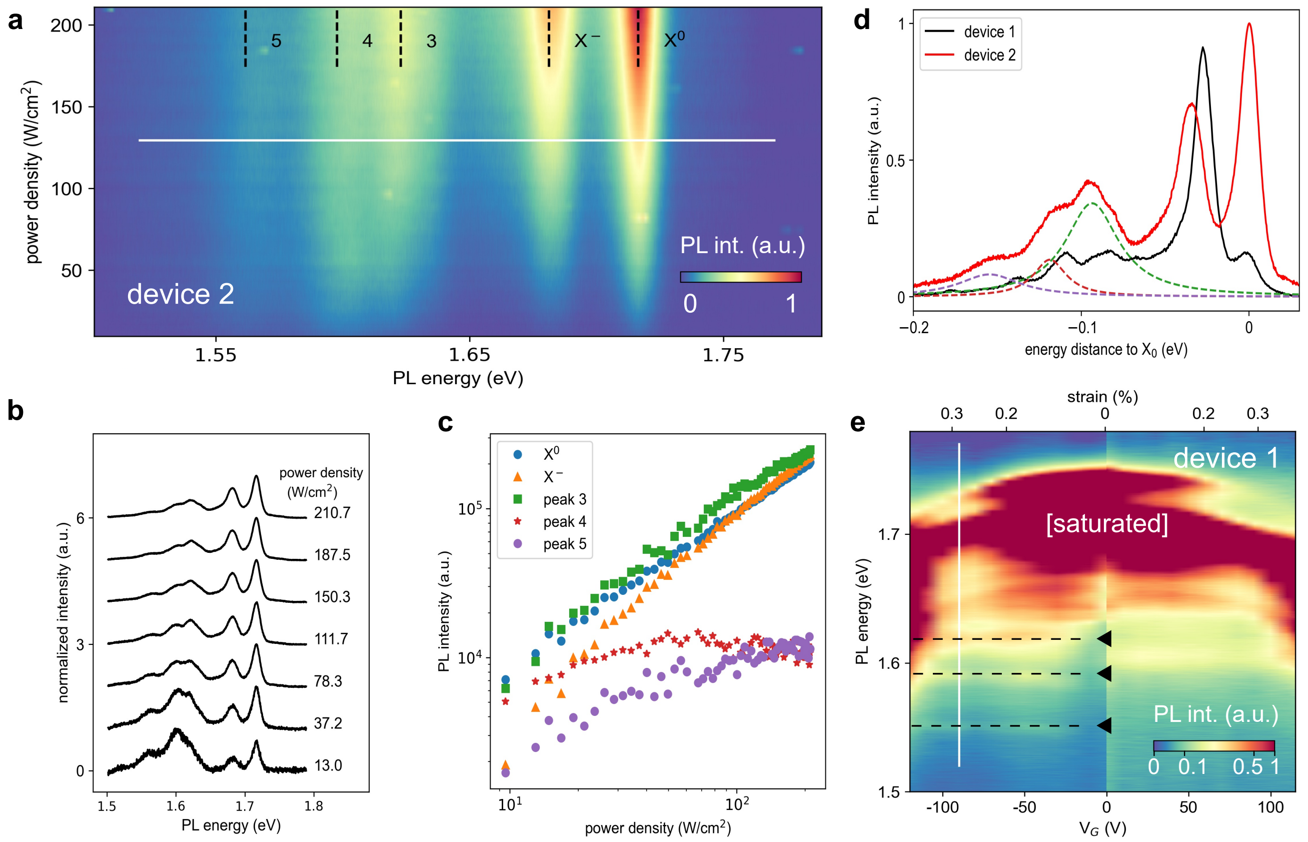

SUPPLEMENTARY FIG. 3. **Defect characterization.** In Figure 3 of the main text we observe in the PL energy range between 1.55-1.6 eV strain-independent lines that we assign to defect-related excitons. This assignment is supported by the detailed analysis of excitation power and V_G dependence of PL spectra. (a) PL vs. excitation power for an unstrained membrane ($V_G = 0$) measured on device 2 at 77K. We identify neutral (X^0) and charged exciton (X^-) and three other peaks labelled 3-5 in decreasing energy order (black dashed lines). (b) PL spectra at selected excitation power densities. Spectra are normalized and offset for clarity. Peaks 3-5 dominate the PL spectrum at lower excitation powers; X^0 and X^- dominate at higher powers. (c) PL integrated intensities of the five peaks marked with dashed lines in a) as a function of excitation power density. PL intensities are obtained by fitting the PL spectra to five Lorentzians. X^0 and X^- show an approximately linear dependence with excitation power. This behaviour is typical for free excitons [5]. PL intensities of peaks 3, 4 and 5 saturate with power indicating the defect-related character of the emission at those energies. (d) PL spectra of devices 1 and 2 featuring the same set of defect peaks, confirming their reproducibility. Spectra for device 1 (black) and device 2 (red) are extracted from the white lines in (e) and (d), respectively. Typically fitted spectra for device 2 corresponding to peaks 3-5 shown in (a)-(c) are plotted with dashed lines. (e) PL spectra vs. V_G for device 1. The energies of defect-related states are almost strain-independent, as expected from modelling. All (a)-(e) are measured at excitation 532 nm; (d) and (e) are measured at excitation power density 250 W/cm^2.

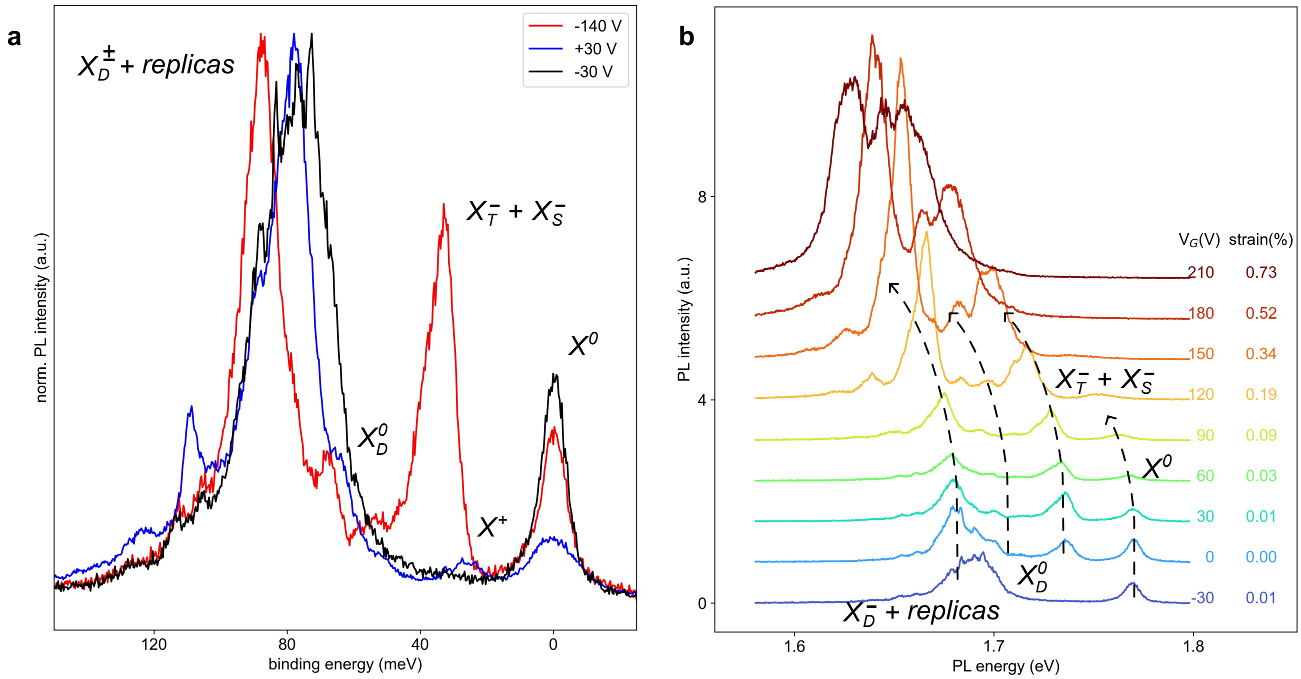

SUPPLEMENTARY FIG. 4. **Strain response of excitonic states in WSe2 at 10 K.** In Figure 5a of the main text we show a colormap of PL vs. VG at 10K measured on device 1. Here we show selected individual PL spectra extracted from the map in Fig. 5a, identify the different excitonic species present in the sample, and track their evolution with increasing strain. (a) Normalized PL spectra in the electron doping (red line), hole doping (blue) and charge neutrality (black) regimes under negligible strain. We identify a bright neutral exciton (X^0) at 1.769 eV, positive (X^+, binding energy 29 meV) and negative trions (X_T^- and X_S^-, 32 and 38 meV) and a dark neutral exciton (X_D^0, 17 meV below X_S^-). We observe a lower energy PL band peaking at 80 meV and 87 meV below X^0 for hole and electron doping, respectively. We assign this PL band to dark charged excitons ($X_D^{\pm}$) and their replicas. (b) Selected PL spectra at different voltages (strain values), offset for clarity. We mark the excitonic species identified in (a) with dashed lines and track their PL signatures upon increasing voltage. PL intensity of dark trions and their replicas and dark neutral exciton increase 6 and 20 times, respectively, as compared to their unstrained values when their energies approach the D1 hybridization energy 1.52 eV.

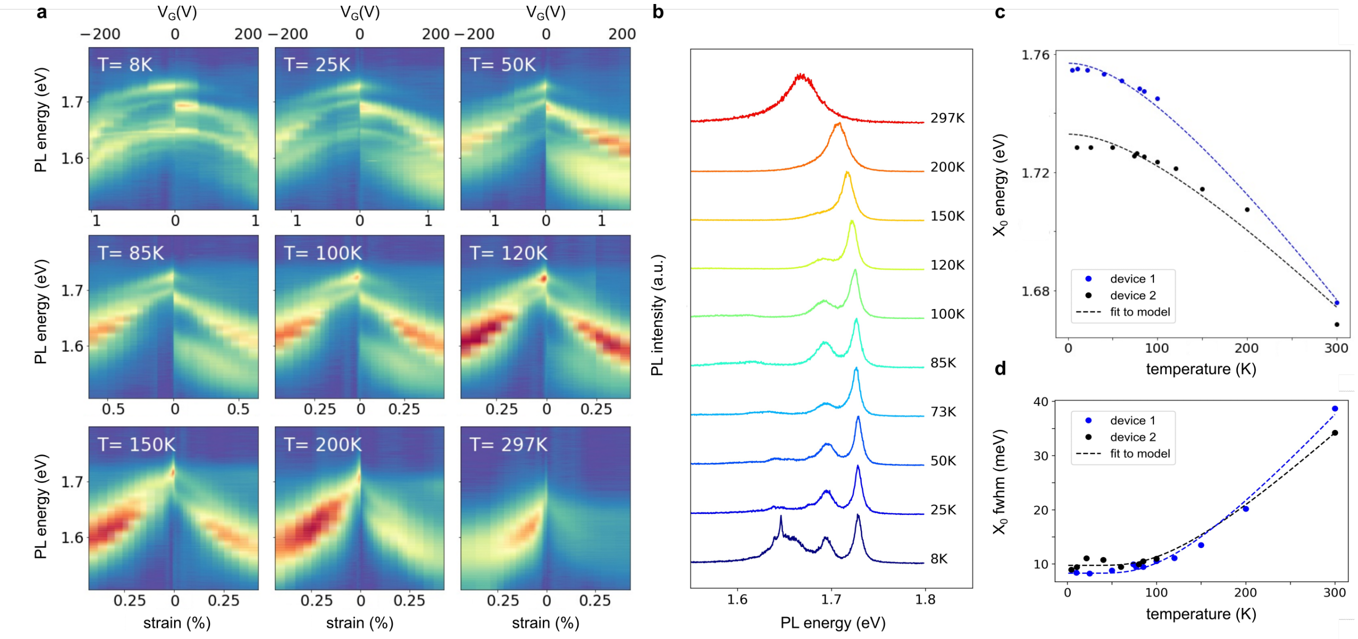

SUPPLEMENTARY FIG. 5. **Temperature-dependent strain response for the full range of measured temperatures.** In Figure 5 of the main text we show PL vs. V_G for three different temperatures, 10 K, 100 K and 300 K. Here we measure on device 2 temperature-dependent PL vs. V_G at nine different temperatures in the 10-300 K range to show the gradual appearance of the features described in the main text. We track neutral exciton position and linewidth as a function of temperature to accurately characterize the temperature of our measurements. (a) PL spectra versus strain maps acquired at different temperatures ranging from 10 K to 297 K. For all temperatures, the gate voltage range is -200 V to 200 V. Note that the strain in the membrane depends on temperature, in addition to V_G, as explained in the main text. The asymmetry in the PL response between the p- and n-doping regimes described in the main text gradually appears for $T > 120$ K. (b) PL spectra for the unstrained membrane ($V_G = 0$) at different temperatures measured on the device of the main text. Spectra are normalized to their respective maximum intensities and upshifted for clarity. (c) Neutral exciton position as a function of temperature for two devices. We compare device 1, shown in the main text, with device 2, shown in (a). Experimental data is fitted to Varshni equation $E_G(T) = E_0 - \frac{\alpha T^2}{T+\beta}$ [6]. From the fit to the Varshni equation we extract the values for the parameters α =4.40 10^{-4}, 3.25 10^{-4} $eV K^{-1}$ and β=199, 200K for devices 1 and 2, respectively. The obtained values are in good agreement with those in the literature [6, 7]. Tracking the blueshift of the neutral exciton energy with decreasing temperatures allows us to monitor the cooling of the samples. (d) Neutral exciton full width half maximum as a function of temperature. Experimental data are fitted to $\Gamma(T) = \Gamma_0 - S < hw > (coth(\frac{<hw>}{2kT}) - 1)$ [6]. The widths of the neutral exciton at low temperatures, 8 meV and 9 meV, confirm the high quality of our samples.

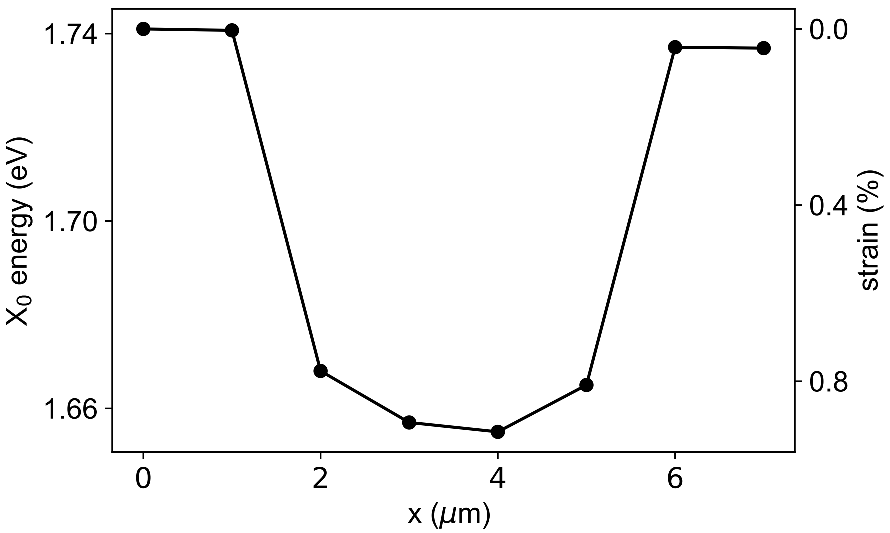

SUPPLEMENTARY FIG. 6. **Strain spatial uniformity.** Neutral exciton PL energy of a suspended WSe_2 monolayer (device 2) at $T = 77\,\mathrm{K}$ and $V_G = -200\,\mathrm{V}$ measured across a linescan through the center of the membrane. The redshift of the neutral exciton energy with respect to its unstrained value is converted to strain on the right axis. We observe sudden jumps of nearly 1% strain around the borders of the $5\mu m-$diameter membrane and small strain gradients ($< 0.07\%/\mu m$) around its center, confirming that the strain is spatially homogeneous within the area probed by our laser spot (800 nm radius).

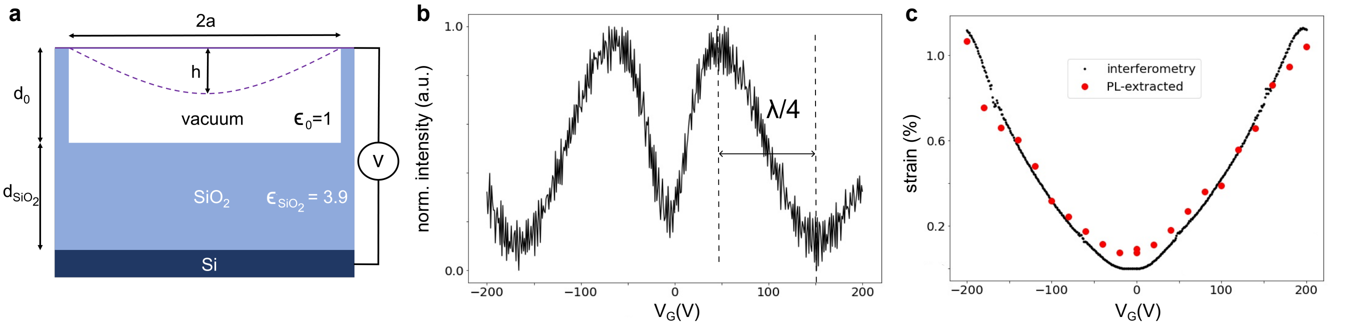

SUPPLEMENTARY FIG. 7. **Interferometric confirmation of strain extraction**. In the main manuscript, we obtain strain at each gate voltage by measuring the shift in the energy position of the neutral exciton compared to its unstrained position. Here, we confirm the assignment of strain via an independent interferometric measurement. (a) Schematic of the device used in the main manuscript seen as an interferometric cavity. A circular monolayer WSe_2 membrane of radius $a = 2.5\,\mu$m is suspended over a hole in SiO_2 and actuated (displacement h) by applying gate voltage V_G between WSe_2 and a Si substrate underneath (see Methods for details). The device is measured at room temperature by recording the reflected laser intensity of the laser beam ($\lambda = 633\,$nm, $P = 10\,\mu$W) focused on the membrane's center vs. V_G (b). The interference of the laser beam in the optical cavity consisting of vacuum, SiO_2 and Si results in the changes of absorption by the membrane. With increasing $|V_G|$, the membrane moves downwards and the reflected intensity changes depending on the position of the membrane with respect to the nodes of the interference pattern. Quantitatively, a displacement of the membrane by $\lambda/4$ produces a change of π (half a period) in the phase of the sinusoidal modulation of the reflected signal. In this way we extract the position of the membrane's center h vs. V_G from the observed intensity of reflected light. Next, we convert the displacement into mechanical strain using the formula $\varepsilon = \frac{2h^2}{3a^2}$ obtained from the analysis of the system's geometry [8]. The resulting data are plotted in c) as black dots. For comparison, in the same graph we plot as red circles the values of strain obtained from the photoluminiscence energetic shift of the excitonic peaks following the same procedure as in the main text. Both approaches to estimate strain produce very similar strain values.

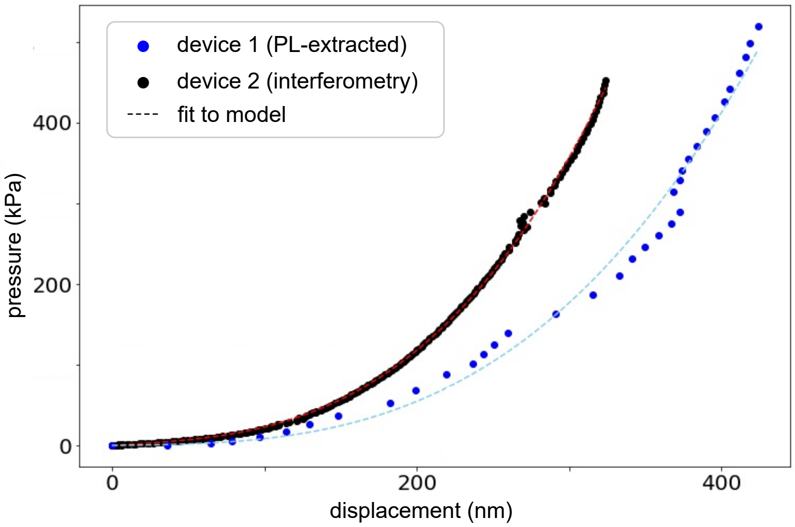

SUPPLEMENTARY FIG. 8. **Extraction of WSe_2 mechanical constants from optical measurements**. To confirm the validity of the model of strain induced by electrostatic force used in the main text, we extract the mechanical constants of WSe_2 from PL measurements at room temperature. To do this, we first calculate the electrostatic pressure P acting on the suspended WSe_2 at a particular gate voltage. Modelling the system as a parallel plate capacitor (WSe_2/Si) with two media inside of it (vacuum, SiO_2), we obtain $P = \frac{\epsilon_0 \epsilon_{SiO_2}^2}{(\epsilon_0 d_{SiO_2} + \epsilon_{SiO_2} d_0)^2} \frac{V_G^2}{2}$. We then calculate the displacement of the central point of the membrane h from strain obtained via PL measurements using $\varepsilon = \frac{2h^2}{3a^2}$, where a is the radius of the membrane. Finally, $P(h)$ data obtained this way are plotted for two different devices 1 and 2. These data are fitted using an analytical model for $P(h)$ that is based on the well-known bulge-test equation in thin-film mechanics [8, 9]. This model describes the deflection of a uniform circular membrane under uniform pressure usblueing Young modulus E_{2D} and built-in strain ε_0 as free parameters as $P = \frac{4E_{2D}\varepsilon_0}{a^2}h + \frac{8E_{2D}}{3(1-\nu)a^4}h^3$, where $\nu = 0.2$ is the Poisson ratio for WSe_2 [10]. The data are described well by the fits (dashed lines). We obtained $E_{2D} \sim 74$ N/m and $\varepsilon_0 = 0.04$ % for device 1 and $E_{2D} \sim 140$ N/m and $\varepsilon_0 = 0.12$ % for device 2. The values are consistent with the mechanical constants typically reported for WSe_2 at room temperature [4].

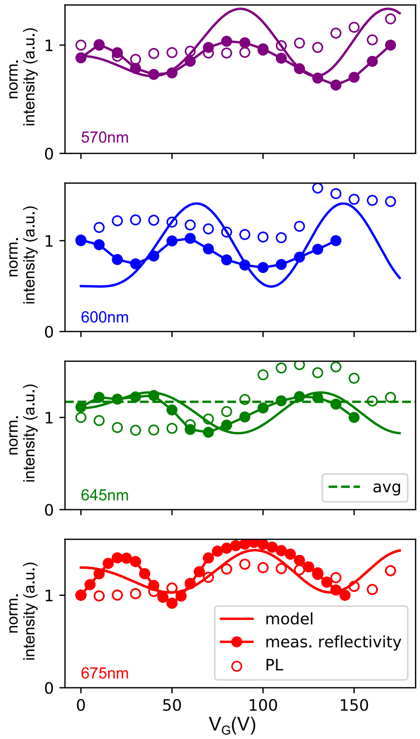

SUPPLEMENTARY FIG. 9. **Quantification of interference effects.** One artefact that can produce non-monotonous changes in the PL intensity vs. V_G – and hence appear similar to the signatures observed in the main manuscript – arises due to optical interference, see Supplementary Note 1. Indeed, optical interference causes the dependence of light intensity absorbed by the membrane on its displacement under electrostatic pressure. We expect the total PL intensity – by which we mean the integrated intensity of all PL peaks peaks in a spectrum – to vary proportionally. Here, we quantify the possible contribution of this effect. We examine the integrated PL intensity vs. V_G (empty symbols) for different excitation wavelengths (570, 600, 645, 675 nm) on device 5 at room temperature. Room temperature PL intensity in this range of V_G is roughly constant with most changes likely arising from interference effects (Fig. 5). Indeed, we see that for all excitations, the variation in the signal remains small, but its phase shifts, as expected for interference. Furthermore, the signal matches the calculated laser intensity at the position of the device vs. V_G for same wavelengths using a simple model based on transfer matrices that captures interference effects (solid lines). In addition, we also plot the normalized reflectivity vs. V_G, measured as in Supplementary Fig. 7 (Curves are upshifted for clarity). As expected, this data are similar to the PL intensity data as the reflectivity signal maxima coincide with the maxima in absorption. We therefore ascribe the modulation signals seen in the figure to interference effects. Note that the maximum modulation due to interference is found for 645 nm (green empty symbols) and it is smaller than a 30 % of the average intensity. This allows us to set 30 % as an upper boundary for the effect of interference in our experimental data.

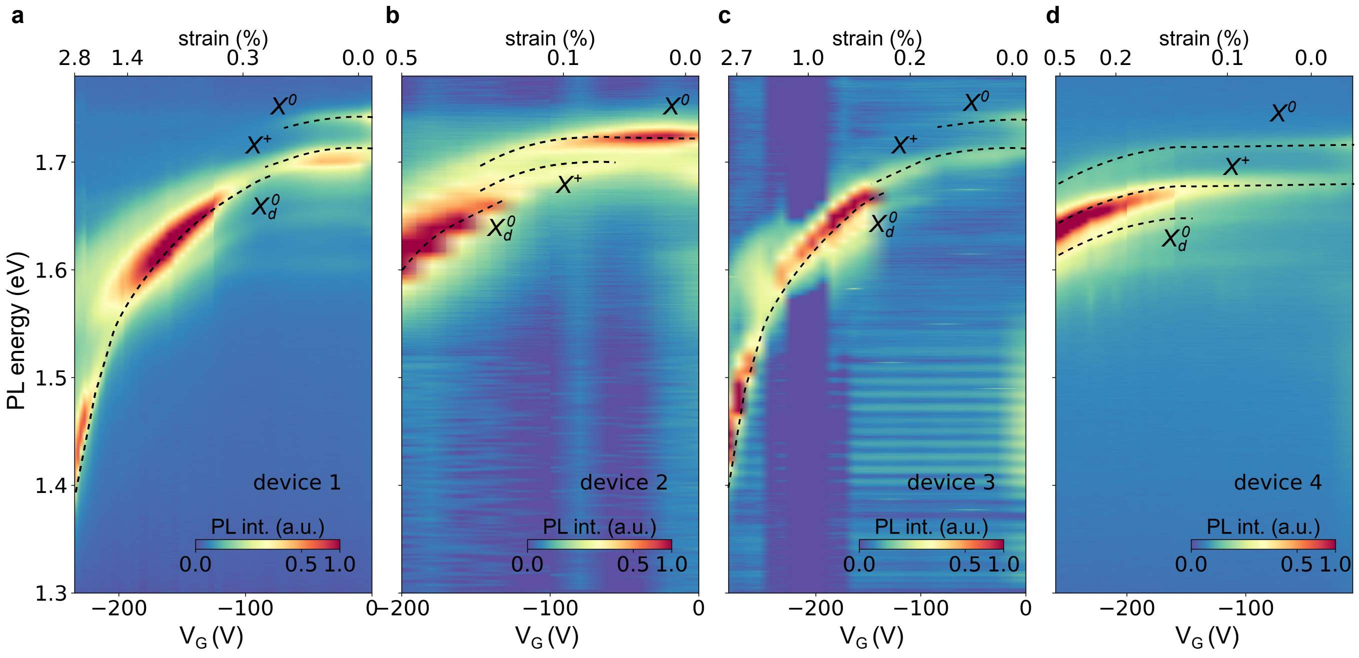

SUPPLEMENTARY FIG. 10. **Repeatability of the data.** PL spectra versus strain maps at 100 K measured on four different samples. In devices 1 and 2 the hole radius is 2500 nm and 3800 nm on devices 3 and 4, hole depth is approximately 660 nm for all devices. All main features reported in the manuscript (strain-dependent excitons and trions, strain-independent defect states, hybridization between defect-related and dark excitons) are observed in all samples. Note different voltage to strain conversion between devices (see also Supplementary Fig. 8). Devices 2 and 4 strain much less efficiently compared to devices 1 and 2, only allowing us to reach the strain levels necessary for the hybridization with D1 (and not D2). The energy positions of large increases in PL (and associated defect states D1 and D2) are device-independent. Horizontal lines between 1.3 eV-1.6 eV are a measuring artifact.

SUPPLEMENTARY NOTE 2: DETAILS OF CALCULATIONS

Tight binding model: In the following, we briefly outline our tight-binding (TB) approach to model strain-dependent photoluminescence of the strained WSe_2 layer. Given the large diameter ($\approx 5\mu m$) of the suspended monolayer in relation to the irradiation spot size of the laser ($\approx 1\mu m$) we assume homogeneous biaxial strain at the optically active monolayer region.

We parametrize our TB model directly from density functional theory (DFT). We perform fully spin-polarized, structural and electronic optimization of the pristine cell with the DFT software package VASP [11–14] and find an energetically favorable lattice constant of $a_0 = 3.322$Å. Plane wave energy cutoff is set to 500 meV and the systems are electronically converged to $\delta E \approx 10^{-9}$ eV. We generate TB models based on maximally localized Wannier functions (MLWFs) from a set of DFT calculations of the pristine system sampling different lattice constants $a = a_0 \cdot (1 + \varepsilon)$ corresponding to different uniform strain. We choose strains $\varepsilon \in [-1\%, 0\%, +1\%, +2\%, +3\%]$. Our calculations include 35Å vacuum perpendicular to the membrane and use a $25 \times 25 \times 1$ Monkhorst-Pack **k**-space grid. The exchange-correlation functional of choice is Perdew-Burke-Ernzerhof (PBE). The ionic positions (for fixed a) are fully relaxed to residual forces less than 10^{-2} eV/Å for each strain value. Stretching the system also induces lateral contraction of the layer thickness (i.e. the distance between the two selenium atoms along the z direction) shrinking by about 1.4% at +2% in-plane strain.

For each converged DFT calculation, we extract TB parameters using wannier90 [15–18] which calculates the maximally localized Wannier functions. We project on (with spin) six p-orbitals on each chalcogen site and ten d-orbitals on each metal site. The disentanglement converges after roughly 500 iterations with window parameters (outer: [−12.0eV, 6eV], inner: [−12.0eV, 0.5eV]) to final spreads between 49 and 46 Å^2 depending on the involved strain. Semi-local DFT severely underestimates the resulting single particle band gaps [19, 20]. We correct this in TB via a scissor operator [21] that results in a band gap of 2.5 eV which agrees with both experimental and many-body GW calculations [22–24]. In addition to the Kohn-Sham Hamiltonian in the Wannier basis, we also calculate the representation of the position operator for subsequent calculations of dipole matrix elements. In order to accurately investigate at intermediate strain values we linearly interpolate the matrix entries of both the periodic Hamiltonian and the periodic position operator between two sampling points ε_a,ε_b (i.e. $\varepsilon_a, \varepsilon_b \in [-1\%, 0\%, +1\%, +2\%, +3\%]$):

$$\begin{aligned} \hat{H}^{\lambda_x,\lambda_y}\varepsilon &= \hat{H}^{\lambda_x,\lambda_y}\lfloor\varepsilon\rfloor + (\varepsilon - \lfloor\varepsilon\rfloor)\Big(\hat{H}^{\lambda_x,\lambda_y}(\lceil\varepsilon\rceil) - \hat{H}^{\lambda_x,\lambda_y}(\lfloor\varepsilon\rfloor)\Big) \\ \hat{r}^{\lambda_x,\lambda_y}\varepsilon &= \hat{r}^{\lambda_x,\lambda_y}\lfloor\varepsilon\rfloor + (\varepsilon - \lfloor\varepsilon\rfloor)\Big(\hat{r}^{\lambda_x,\lambda_y}(\lceil\varepsilon\rceil) - \hat{r}^{\lambda_x,\lambda_y}(\lfloor\varepsilon\rfloor)\Big) \end{aligned} \quad (1)$$

We have taken care to ensure that the Wannier bases of the different DFT sampling points are converged to consistent solutions that allow for meaningful interpolation. This interpolation allows us to efficiently calculate the band structure of pristine WSe_2 for arbitrary strains (up to about 3%, see Supplementary Fig. 11).

The influence of strain is different for the conduction and valence bands and also **k**-dependent. Tensile strain predominantly causes the conduction band minima at K/K' to shift downwards in energy. The strongly spin-split valence band also lowers in energy around K/K', but does so at a much lower rate. The competing minimum of the conduction band at Q (indirect band gap) seems mostly unaffected by strain and becomes increasingly energetically separated from the K/K' minimum at larger tensile strains.

PL calculations: We model the intensity variations of the PL peaks with increasing strain as single particle effects: strain causes the bulk conduction bands to shift downwards in energy, causing an energetic overlap with localized defect levels. The resulting hybridization results in a substantial increase in oscillator strength. In order to probe this hypothesis we study optical transitions between the relevant bands in a defect supercell. Comparison between different vacancies (Se-vacancy, Se-divacancy, W-vacancy) strongly favours the simple Se-vacancy due to both number and energy of midgap defect levels (see Supplementary Fig. 13). The selenium mono vacancy features two defect levels (four with spin) with shallow donor character. Conduction, defect and valence bands all shift downwards in energy under tensile strain but do so at different rates (vb: −74 meV/%, D1: −71 meV/%, D2: −73 meV/%, cb: −182 meV/%). The spin split conduction bands then undergo a sequence of avoided crossings with the two defect levels $D1$ and $D2$.

We expect optical transitions to be the dominant decay channel for the excitons in our system (cryostat temperatures) and thus calculate dipole matrix elements

$$|\mathbf{J} \cdot \langle \Psi^v | \hat{\mathbf{r}} | \Psi^c \rangle|^2 \quad (2)$$

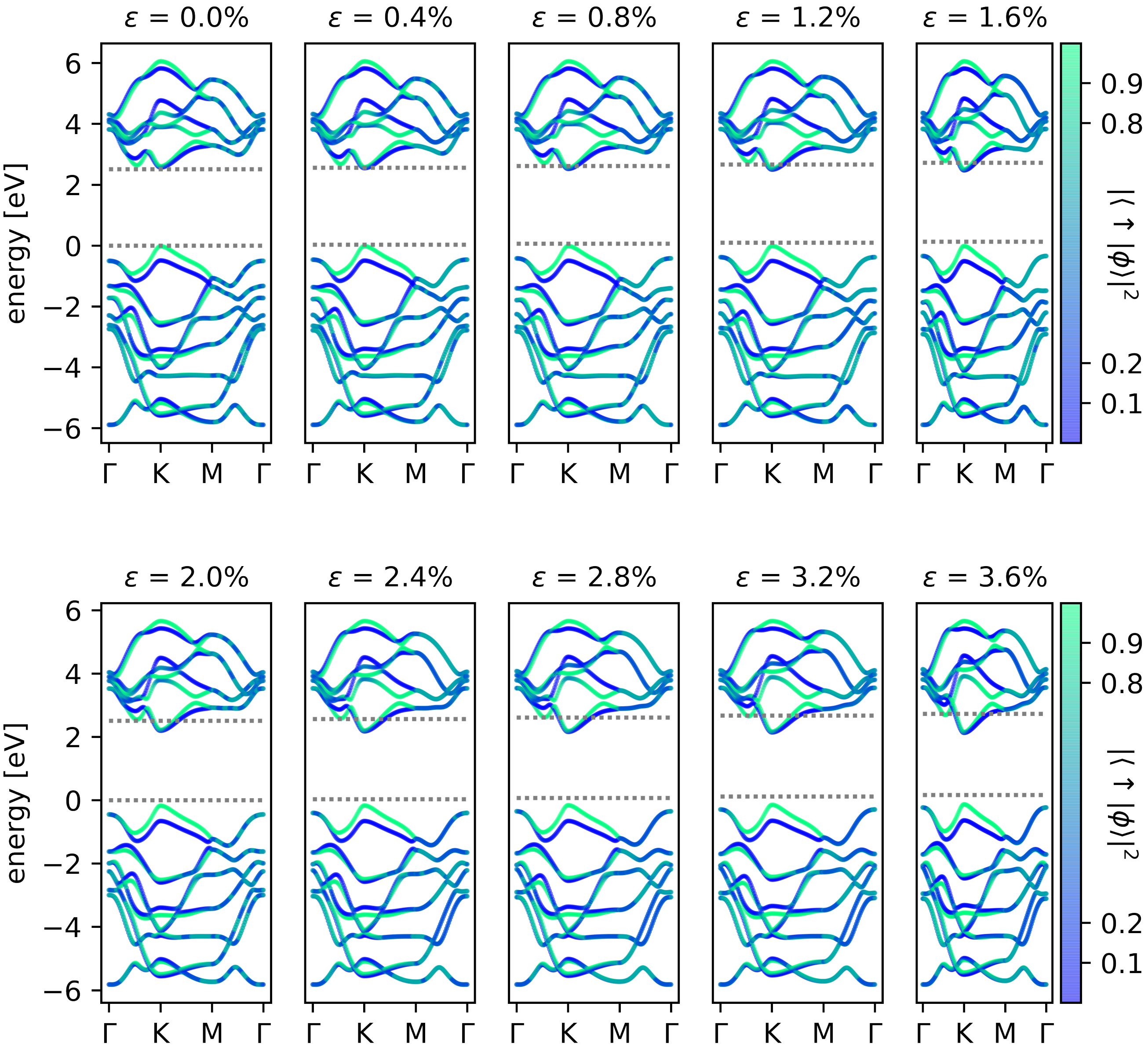

SUPPLEMENTARY FIG. 11. **Band structure of pristine WSe_2 calculated from TB Hamiltonians**. Color indicates spin-up (↑) projection of the underlying Bloch states ϕ. Band gaps for all strains are corrected by about 1.2 eV via scissor operators.

with topmost valence wavefunction $|\Psi^v\rangle$ and conduction wave function $|\Psi^c\rangle$ (this nomenclature counts defect levels as conduction bands) and polarization vector $\mathbf{J}$. We extract the position operator from Wannier90 [15–18]:

$$\langle \mathbf{R}, n|\hat{\mathbf{r}}|\mathbf{0}, m\rangle = \mathrm{i}\frac{V}{(2\pi)^3}\int e^{\mathrm{i}\mathbf{k}\cdot\mathbf{r}}\langle n, \mathbf{k}|\nabla_{\mathbf{k}}|m, \mathbf{k}\rangle d\mathbf{k} \tag{3}$$

Our considerations are limited to the direct band gap at the K point. We also consider the possibility of momentum relaxation at the lattice defect (non radiative transition from K to K') followed by radiative decay into the valence band which features opposite spin polarization in the other valley. This is incorporated by an overlap integral between

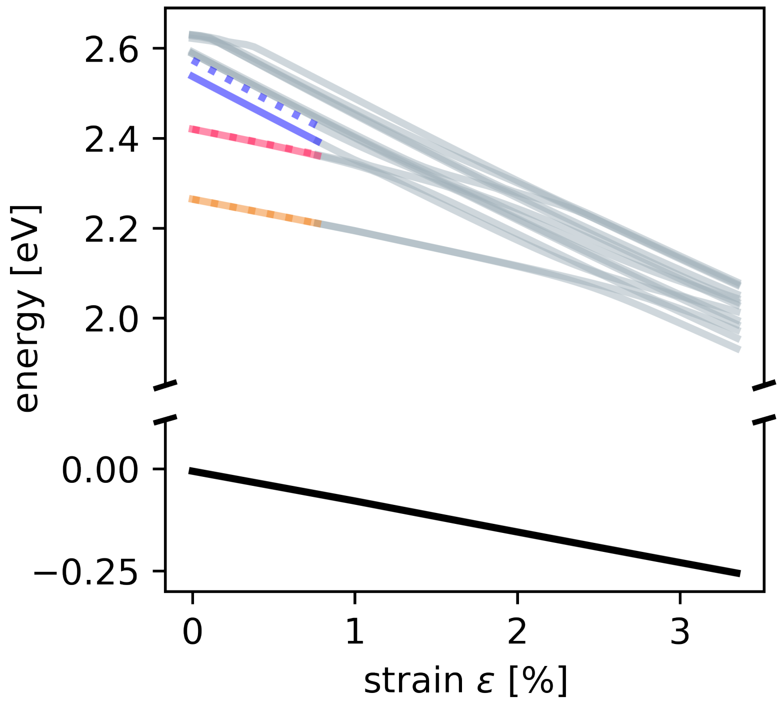

SUPPLEMENTARY FIG. 12. **Band evolution with strain.** Evolution of topmost valence band (solid black line, only one spin component shown), D1 (red lines, solid and dotted correspond to spin components), D2 (orange lines) and bottom of the spin split conduction band (blue lines). Coloring valid for strains up to 0.8%. For higher strains the bands start to continuously intersect and are colored grey.

almost degenerate (to within ±2.5 meV) states at the two valleys.

$$|\mathbf{J}\cdot\langle\Psi^v_{\mathbf{K}}|\hat{\mathbf{r}}(\mathbf{K})|\Psi^c_{\mathbf{K}}\rangle|^2\times 1+\sum_{c',\varepsilon^{c'}_{K'}\approx\varepsilon^c_K}|\langle\Psi^c_{\mathbf{K}}|\Psi^{c'}_{\mathbf{K}'}\rangle|^2 \tag{4}$$

In order to compare with experimental data we calculate PL intensity (oscillator strength, see Eq. 2) as a function of PL energy $\Delta E_{c\to v}$ and strain ε. We shift our PL energy axis by an exciton binding energy of 0.8 eV for all conduction-valence band transitions [25]. Since we have a fully spin resolved TB model we can clearly capture the relative intensity of the bright and dark excitonic features (see Supplementary Fig. 14). Since our model only calculates oscillator strength and does not in fact model the occupation of the corresponding energy bands we see quite pronounced horizontal features at the defect energies. These should however only become effectively populated (and thus visible) once the conduction bands approach their energy due to strain. At the strain values where the dark conduction band produces avoided crossings with the defect levels hybridization occurs and the PL intensity forms peaks since momentum and spin selection rules no longer fully extinguish the PL signal.

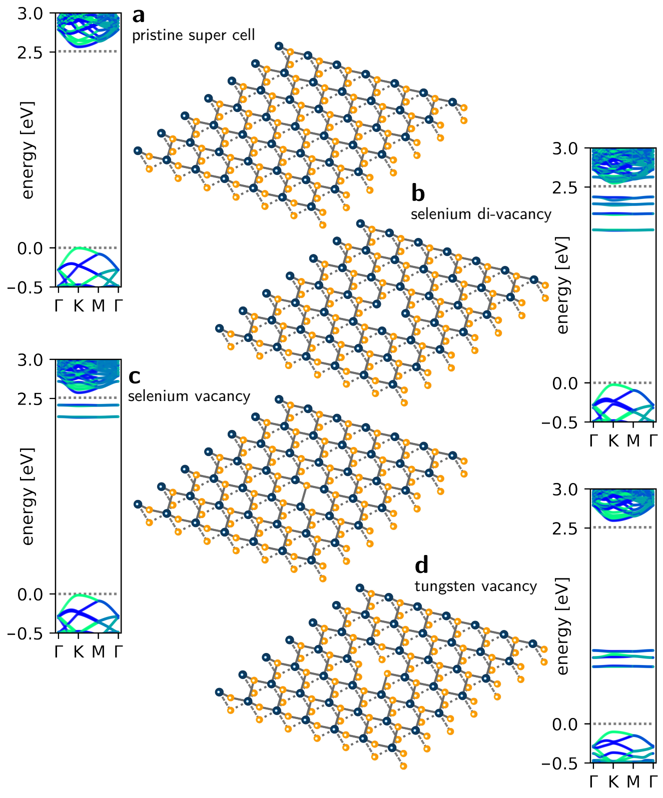

SUPPLEMENTARY FIG. 13. **Defect energies.** Tight binding band structure along $\Gamma KM\Gamma$ and lattice plots for 7×7 super cells of tungsten diselenide for **a** pristine reference, **b** selenium di-vacancy defect, **c** selenium mono-vacancy defect, **d** tungsten vacancy. Horizontal dotted lines serve as guide to the eye for the pristine band gap with scissor correction applied.

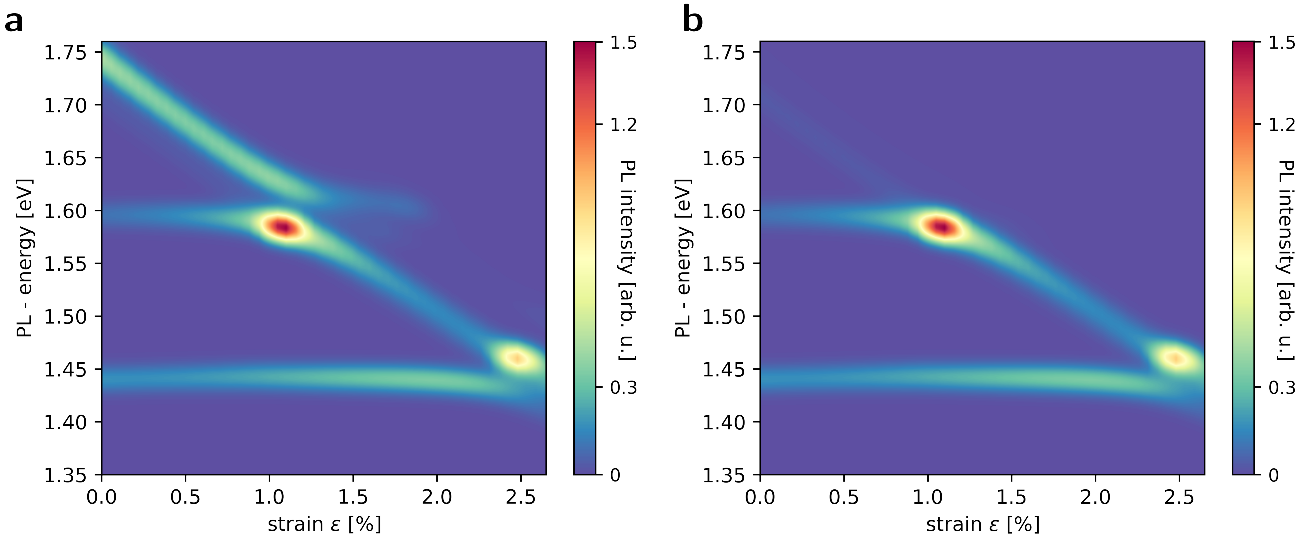

SUPPLEMENTARY FIG. 14. **PL intensity as a function of PL energy and strain.** Modeled PL intensity for a 17×17 Se single vacancy super cell in WSe_2 (i.e. the expression of transition matrix elements in 4 evaluated at K and K') plotted as a function of strain ϵ and PL energy.**a** no relative weighting of bright and dark feature, **b** bright feature suppressed by a factor of $e^{-(36meV/8.6meV)}\approx 1/66$ (to account for thermal population differences). We include a Gaussian smearing of width 10 meV for all energies.

[1] Liu, E. *et al.* Gate tunable dark trions in monolayer wse2. *Physical Review Letters* **123**, 027401 (2019).

[2] Li, Y. *et al.* Measurement of the optical dielectric function of monolayer transition-metal dichalcogenides: Mos2, mose2, ws2, and wse2. *Physical Review B* **90**, 205422 (2014).

[3] Aslan, O. B., Deng, M. & Heinz, T. F. Strain tuning of excitons in monolayer wse2. *Physical Review B* **98**, 115308 (2018).

[4] Aslan, B. *et al.* Excitons in strained and suspended monolayer wse2. *2D Materials* **9**, 015002 (2021).

[5] Schmidt, T., Lischka, K. & Zulehner, W. Excitation-power dependence of the near-band-edge photoluminescence of semiconductors. *Physical Review B* **45**, 8989–8994 (1992).

[6] Arora, A. *et al.* Excitonic resonances in thin films of wse2: from monolayer to bulk material. *Nanoscale* **7**, 10421–10429 (2015).

[7] Huang, J., Hoang, T. B. & Mikkelsen, M. H. Probing the origin of excitonic states in monolayer WSe2. *Scientific Reports* **6** (2016).

[8] Nicholl, R. J. *et al.* The effect of intrinsic crumpling on the mechanics of free-standing graphene. *Nature Communications* **6** (2015).

[9] Merle, B. *Mechanical Properties of Thin Films Studied by Bulge Testing*. Ph.D. thesis (2013).

[10] Kang, J., Tongay, S., Zhou, J., Li, J. & Wu, J. Band offsets and heterostructures of two-dimensional semiconductors. *Applied Physics Letters* **102**, 012111 (2013).

[11] Kresse, G. & Furthmüller, J. Efficient iterative schemes for ab initio total-energy calculations using a plane-wave basis set. *Physical Review B* **54**, 11169–11186 (1996).

[12] Kresse, G. & Furthmüller, J. Efficiency of ab-initio total energy calculations for metals and semiconductors using a plane-wave basis set. *Computational Materials Science* **6**, 15–50 (1996).

[13] Kresse, G. & Hafner, J. Ab initio molecular dynamics for liquid metals. *Physical Review B* **47**, 558–561 (1993).

[14] Kresse, G. & Hafner, J. Ab initio molecular-dynamics simulation of the liquid-metal–amorphous-semiconductor transition in germanium. *Physical Review B* **49**, 14251–14269 (1994).

[15] Pizzi, G. *et al.* Wannier90 as a community code: new features and applications. *Journal of Physics: Condensed Matter* **32**, 165902 (2020).

[16] Marzari, N., Mostofi, A. A., Yates, J. R., Souza, I. & Vanderbilt, D. Maximally localized wannier functions: Theory and applications. *Reviews of Modern Physics* **84**, 1419–1475 (2012).

[17] Souza, I., Marzari, N. & Vanderbilt, D. Maximally localized wannier functions for entangled energy bands. *Physical Review B* **65**, 035109 (2001).

[18] Mostofi, A. A. *et al.* An updated version of wannier90: A tool for obtaining maximally-localised wannier functions. *Computer Physics Communications* **185**, 2309–2310 (2014).

[19] Kormányos, A. *et al.* k·p theory for two-dimensional transition metal dichalcogenide semiconductors. *2D Materials* **2**, 022001 (2015).

[20] Khatibi, Z. *et al.* Impact of strain on the excitonic linewidth in transition metal dichalcogenides. *2D Materials* **6**, 015015 (2018).

[21] Levine, Z. H. & Allan, D. C. Linear optical response in silicon and germanium including self-energy effects. *Physical Review Letters* **63**, 1719–1722 (1989).

[22] Shi, H., Pan, H., Zhang, Y.-W. & Yakobson, B. I. Quasiparticle band structures and optical properties of strained monolayer mos2 and ws2. *Physical Review B* **87**, 155304 (2013).

[23] Ugeda, M. M. *et al.* Giant bandgap renormalization and excitonic effects in a monolayer transition metal dichalcogenide semiconductor. *Nature Materials* **13**, 1091–1095 (2014).

[24] Deilmann, T. & Thygesen, K. S. Finite-momentum exciton landscape in mono- and bilayer transition metal dichalcogenides. *2D Materials* **6**, 035003 (2019).

[25] Hanbicki, A., Currie, M., Kioseoglou, G., Friedman, A. & Jonker, B. Measurement of high exciton binding energy in the monolayer transition-metal dichalcogenides WS2 and WSe2. *Solid State Communications* **203**, 16–20 (2015).
